# Systematic review of the use of big data to improve surgery in low‐ and middle‐income countries

**DOI:** 10.1002/bjs.11052

**Published:** 2019-01-08

**Authors:** S. R. Knight, R. Ots, M. Maimbo, T. M. Drake, C. J. Fairfield, E. M. Harrison

**Affiliations:** ^1^ Surgical Informatics, Centre for Medical Informatics, Royal Infirmary of Edinburgh University of Edinburgh Edinburgh UK; ^2^ Department of General Surgery Kitwe Teaching Hospital Kitwe Zambia

## Abstract

**Background:**

Technological advances have led to the generation of large amounts of data, both in surgical research and practice. Despite this, it is unclear how much originates in low‐ and middle‐income countries (LMICs) and what barriers exist to the use of such data in improving surgical care. The aim of this review was to capture the extent and impact of programmes that use large volumes of patient data on surgical care in LMICs.

**Methods:**

A PRISMA‐compliant systematic literature review of PubMed, Embase and Google Scholar was performed in August 2018. Prospective studies collecting large volumes of patient‐level data within LMIC settings were included and evaluated qualitatively.

**Results:**

A total of 68 studies were included from 71 LMICs, involving 708 032 patients. The number of patients in included studies varied widely (from 335 to 428 346), with 25 reporting data on 3000 or more LMIC patients. Patient inclusion in large‐data studies in LMICs has increased dramatically since 2015. Studies predominantly involved Brazil, China, India and Thailand, with low patient numbers from Africa and Latin America. Outcomes after surgery were commonly the focus (33 studies); very few large studies looked at access to surgical care or patient expenditure. The use of large data sets specifically to improve surgical outcomes in LMICs is currently limited.

**Conclusion:**

Large volumes of data are becoming more common and provide a strong foundation for continuing investigation. Future studies should address questions more specific to surgery.

## Introduction

**Figure 1:**
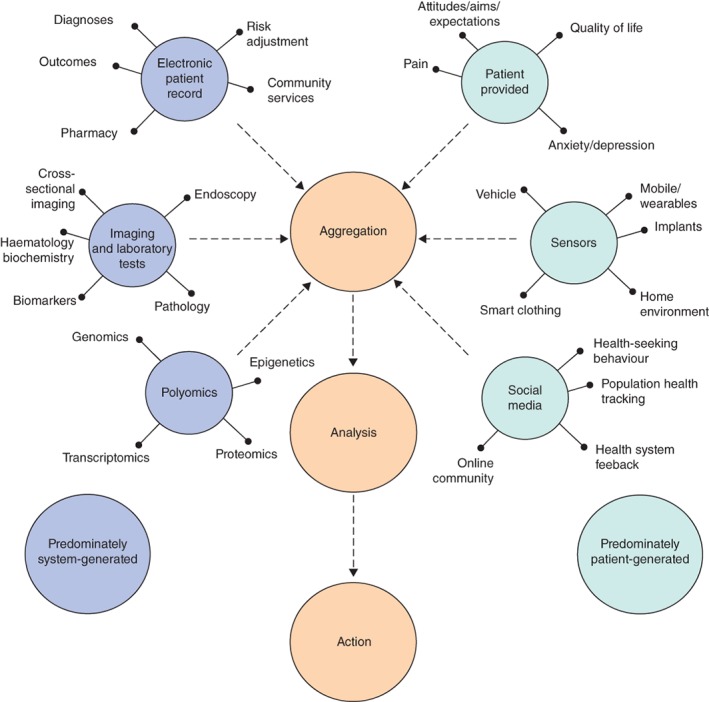
Conceptualizing big data in healthcare. Health system data are aggregated with data generated by the individual and their environment. Data are transformed and analysed to generate actionable output

The concept of ‘big data’ describes the use of unstructured digital information, usually from multiple sources, that is often collected with no clearly defined purpose for future use^1^. The volume of data already being produced is vast, with frequent increases in complexity, variety and speed^2^. Big data in surgery can be defined as the amalgamation and integration of various data sources along the patient pathway to produce a rich matched data set^3^ (*Fig*. 1).

The analysis and translation of big data to maximize quality and improve patient care is a priority for healthcare systems^4^. It is envisaged that measurement and modelling of patient health states and outcomes will quickly become the biggest driver of best practice and healthcare policy^5^. Continual analysis of patient‐level outcomes has already been demonstrated to significantly reduce morbidity and mortality in high‐income countries^6^.

However, discussions around large‐volume patient data frequently place little emphasis on their application in low‐ and middle‐income countries (LMICs), despite the potential for vast gains in patient outcomes and surgical service quality^7^. Currently, LMICs may lack the ability to gather reliable data^6^, with an expectation that this situation is unlikely to change in the near future^8^, ^9^. Ensuring that LMICs can keep up to date with technological advances will help to prevent future global health inequalities worsening^10^.

The aim of this review was to evaluate the current applications of large‐volume patient‐level data in surgery in LMICs, together with highlighting where further focus is required to improve outcomes, define quality indicators and achieve universally available safe surgery.

## Methods

An electronic systematic search of the PubMed, Embase and Google Scholar databases was performed in accordance with the PRISMA guidelines^11^, involving all published literature up to the last search on 23 August 2018. The PROSPERO international systematic review registry^12^ was searched to ensure a similar review had not been performed previously and the protocol was registered accordingly (CRD42018108203).

A search of Embase and PubMed was undertaken using the keywords ‘surgery or surg*’, ‘big data’, ‘large data’, ‘informatics’, ‘database’, ‘cohort’ and ‘registry’, combined with LMIC filters as specified by the Cochrane library^13^. Search terms are listed in *Appendix S1* (supporting information). A further supplementary search of Google Scholar was also undertaken. Search limits applied were English language, full text, humans and articles published from 2008 onwards to provide contemporary studies that were likely reflective of current approaches to data capture.

The inclusion criteria were: prospectively collected data (or retrospective analysis of such data) on patients undergoing surgery with care being provided, at least in part, in a LMIC, defined according to the World Bank classification^14^. Studies were excluded if they contained fewer than 100 patients or were RCTs. Conference abstracts were screened to assist in identifying related full‐text articles. Where more than one article was published from a single data set, the article analysing the largest cohort of patients was included.

Following the literature search, article titles were screened by four investigators and those meeting the inclusion criteria were screened further by abstract and then full text as appropriate. Any disagreements were resolved by consensus within the group. Bibliographies from included articles were hand‐searched to identify any further relevant articles.

Data were extracted independently using a standardized pro forma, including year of publication, countries involved in the study, number of patients for each LMIC, patient‐level data type (cohort, database or registry), surgical specialty and measured outcome(s). In multinational studies where the number of patients for individual countries was not reported, the number of patients in the study was recorded. These studies were excluded from analysis mapping of the global distribution of patients across included studies to avoid data skewing. Individual LMICs where there were fewer than 100 patients in multinational studies were also excluded from analysis mapping. However, studies that did not report patient numbers for individual LMICs and all data from multinational studies were included in all other analyses.

Data types were defined as follows: cohort – collection of patient‐level data over a defined short period; database – concerted and long‐term collection of patient‐level data of consecutive patients over a small geographical area; or registry *–* studies meeting database classification but performed over a wide geographical area (such as national registries).

Definitions were discussed and consensus reached within the group where doubt existed regarding particular studies. Owing to the narrative nature of the review, a qualitative analysis was performed using the R statistical program (https://www.R‐project.org/) and the tidyverse package^15^. All analyses and graphical representation of the data can be found at https://argoshare.is.ed.ac.uk/bigdata_review.

## Results

**Figure 2:**
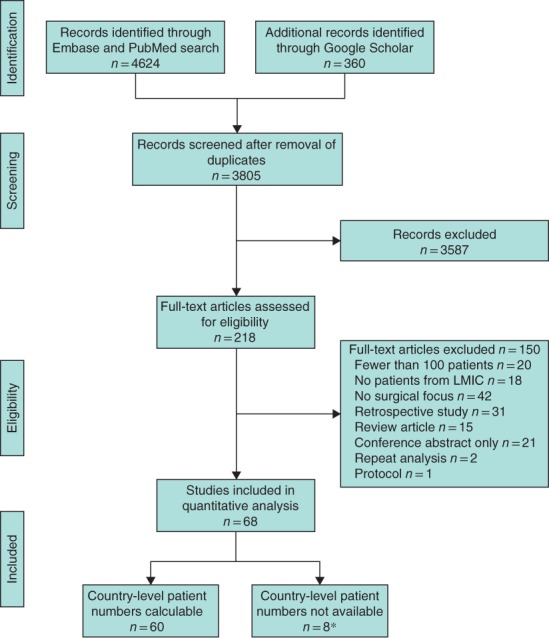
PRISMA flow chart showing selection of studies for review. *Two of these studies provided total LMIC patient number. LMIC, low‐ and middle‐income country

The literature search identified 3805 articles, of which 218 full texts were assessed for eligibility (*Fig*. 2). Following assessment, 68 articles^16^, ^17^, ^18^, ^19^, ^20^, ^21^, ^22^, ^23^, ^24^, ^25^, ^26^, ^27^, ^28^, ^29^, ^30^, ^31^, ^32^, ^33^, ^34^, ^35^, ^36^, ^37^, ^38^, ^39^, ^40^, ^41^, ^42^, ^43^, ^44^, ^45^, ^46^, ^47^, ^48^, ^49^, ^50^, ^51^, ^52^, ^53^, ^54^, ^55^, ^56^, ^57^, ^58^, ^59^, ^60^, ^61^, ^62^, ^63^, ^64^, ^65^, ^66^, ^67^, ^68^, ^69^, ^70^, ^71^, ^72^, ^73^, ^74^, ^75^, ^76^, ^77^, ^78^, ^79^, ^80^, ^81^, ^82^, ^83^, involving 708 032 patients across 71 LMICs, were included in the review (*Tables S1* and *S2*, supporting information). Country‐specific patient numbers were reported in 60 studies but were absent from six^50^, ^51^, ^52^, ^57^, ^62^, ^83^ and two^33^, ^55^ provided total LMIC patient numbers only.

### Patients and studies

**Figure 3:**
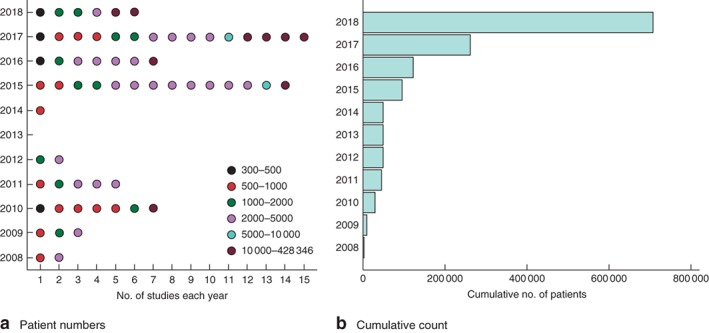
Patient numbers over time in included studies. **a** Patient numbers in each year; **b** cumulative count by year

Studies using big data were well represented across the 10‐year analysis period; however, a dramatic increase in study and patient numbers was seen from 2015 onwards (*Fig*. 3 *a*). Relatively few studies were found for the interval 2012–2014 despite no decrease in the total number of studies returned in the initial literature search (339, 358 and 469 studies in 2012, 2013 and 2014 respectively, compared with a median of 222 (range 129–487) for other years).

The number of patients in the included studies ranged from 335 to 428 346, with a median of 2483 per study. Over 3000 patients were included in 25 of 68 studies; the biggest studies were published in the interval 2015–2018. Studies based on database and registry data were most common and represented 43 of 68 included studies. The majority of data sets identified arose from prospective cohorts of patients. Several of these studies were performed in single centres^43^, ^65^ or single nations^79^, ^81^, with comparisons made with high‐income countries. The largest cohort of patients originated from the DATASUS registry in Brazil (428 346 patients), which explored outcomes after hysterectomy^80^. Five multinational observational cohort studies^50^, ^51^, ^57^, ^82^, ^83^ were performed in the past 5 years, with the majority conducted over 7 days.

### Geographical distribution

**Figure 4:**
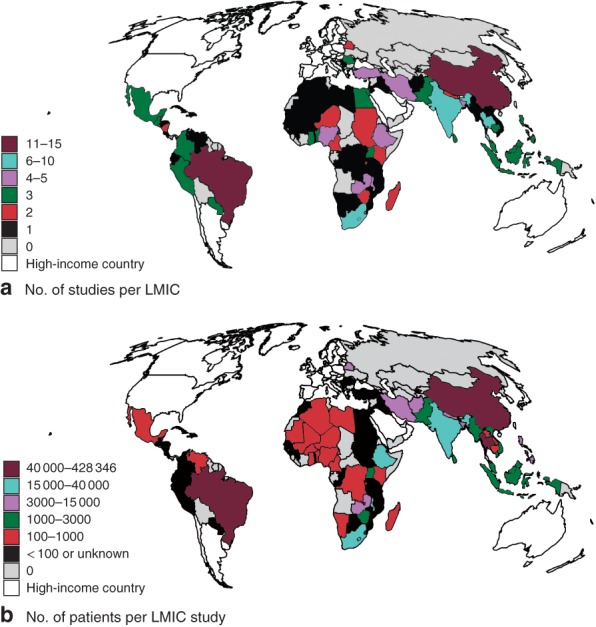
Global distribution of patients and studies across low‐ and middle‐income countries (LMICs) in included articles (2008 to present). **a** Number of studies and **b** number of patients in studies of LMICs. Countries with fewer than 100 patients recruited for a multinational study were excluded from **a**, as were studies in which LMIC‐specific patient numbers were not specified^48^, ^49^, ^50^, ^55^, ^60^, ^81^

The studies had a wide geographical LMIC distribution. The majority, however, were from Brazil (12), China (11), India (5) and Thailand (4) (*Fig*. 4 *a*). Patient‐level data were collected from 71 LMICs in total; overall, patient representation was particularly low in Africa and Latin America (*Fig*. 4 *b*).

### Subject of studies

**Figure 5:**
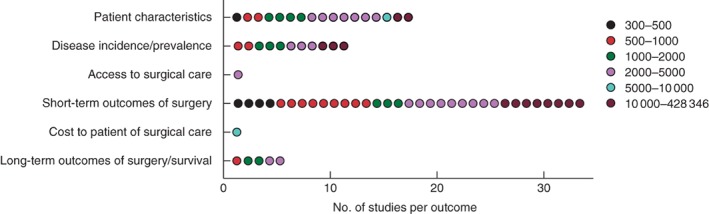
Subject area of large‐volume studies of surgery in low‐ and middle‐income countries in relation to number of study patients

The focus of study varied across included articles (*Fig*. 5). Short‐term outcomes of surgery were most commonly captured (33 studies) and, of these studies, eight included over 10 000 patients each.

Outcomes following cancer surgery were common topics, including breast^19^, ^31^, ^38^, ^45^, ^46^, ^47^, ^77^, gastric^16^, ^22^, ^23^, ^61^, colorectal^24^, ^59^, ^76^, ^81^ and prostate^18^, ^30^ cancer, and hepatocellular carcinoma^56^, ^60^. Cardiac surgery^34^, ^43^, ^65^, ^70^, caesarean section^44^, ^49^, ^69^ and genitourinary fistula^27^, ^33^, ^74^ were also well represented in included articles, whereas clinical presentations included burn management^55^, trauma^66^, appendicitis^71^, groin hernias^35^ and orthopaedic fracture management^48^, ^73^.

However, the overall journey of a patient through the surgical care process was poorly represented, with only a single study^53^ examining access to surgical care and the cost of surgical care to the patient. No study assessed whether the results of big data analyses have resulted in meaningful changes to healthcare systems or had a significant impact on patient outcomes in LMIC settings.

A number of studies successfully demonstrated the ability to assemble large prospective data sets on patients across multiple nations. The International Surgical Outcomes Study^51^ included 15 806 patients in eight LMICs, and the African Surgical Outcomes Study^82^ included 11 422 patients across 25 African countries. These studies captured mortality and complication rates but, as importantly, were able to capture patient risk profiles and patterns of surgical practice. Highlighting differences in surgical outcome by country‐income level, a lack of critical care provision in LMICs was postulated to significantly influence the ability to rescue patients from complications, with implications for resource planning at a governmental level^40^, ^51^, ^82^.

Multinational studies also targeted specific disease areas (GlobalSurg 1: emergency abdominal surgery)^57^ or specific complications of surgery (GlobalSurg 2: surgical‐site infection)^83^. These two studies^57^, ^83^ gathered prospective data on 23 284 patients and demonstrated that low‐income countries carry a disproportionately higher burden of surgical‐site infection and threefold higher mortality rates.

## Discussion

The past 5 years has seen an exponential rise in the number of patients included in studies from LMICs, with some very large cohorts in countries such as Brazil, China and India. Geographical disparities are apparent and are particularly obvious in Africa, where far fewer large studies have been published. The focus is predominately on short‐term outcomes after surgery, together with the epidemiology of diseases commonly treated by surgery. Few studies have focused on the specific needs of resource‐poor environments. It is perhaps too early to determine any positive effects of such work on outcomes in populations of individuals receiving surgical care.

The use of big data to capture patient‐level outcomes in an LMIC setting has increased exponentially over the past 10 years. However, in global cohort studies the proportion of patients recruited from high‐income countries remains much greater^51^, ^57^, ^83^. This may suggest the limiting role of infrastructure and resources within LMICs in collecting patient data. Huge disparity with big data applications currently exists globally; no included studies used big data algorithms to identify patient management, predict outcome or direct healthcare policy.

In high‐income settings, big data are currently the focus of genomewide data analysis^84^, developing personal omics profiles^85^ and individualized oncology treatment^86^. Meanwhile machine‐learning algorithms are being developed to help deliver care, inform health policy and reduce waste^87^, ^88^, ^89^. Technological infrastructure, specialized analytical skills and personal tracking of health statistics using smart phones, particularly in America, is enabling the amalgamation and analysis of big data from multiple sources on an individual level to offer personalized healthcare packages^90^. However, real‐time mobile technology application to measure infectious disease outbreaks in LMICs has been realised^91^, ^92^, and efforts to develop and incorporate multiple levels of patient data should now be a focus.

Combining data from multiple sources to draw population‐level conclusions worldwide is epitomized by the Global Burden of Disease project by the Institute for Health Metrics and Evaluation at the University of Washington. This is a global effort to examine comprehensively the prevalence, incidence and impact of multiple diseases and environmental factors using an extensive network of more than 2500 collaborators from 133 countries^93^. Recent publications include global predictions on cancer burden^94^, child mortality^95^, causes of adult disability^96^ and alcohol use^97^.

Such projects require accurate national data, which do not exist in many regions. National registries can be expensive to establish and run, but are becoming more common in middle‐income countries, such as the Chinese Guangzhou Occupational Cohort^98^ and the Brazilian DATASUS registry^80^.

Comprehensive patient‐level databases or registries are yet to be adopted in the majority of LMICs. Barriers limiting broad adoption include a lack of resources and infrastructure, such as electricity and reliable internet connectivity, combined with skill shortages in medical informatics. The advent of the electronic patient record (EPR) may present the best opportunity for routine data analysis at a health‐system level^99^. Although the costs of set‐up and maintenance can be a barrier, multiple open‐source EPRs now exist which can potentially alleviate some of these^100^. Recently, Rwanda announced the roll‐out of the OpenMRS system to 250 clinics and hospitals across the country^101^. This will bring EPRs into national practice and offer the opportunity for real‐time data collection within a healthcare system to be used for infrastructure planning and research.

Linked to this is the explosion in mobile phone technology. Three‐quarters of the population of sub‐Saharan Africa already lives in an area with mobile internet connectivity^102^. On‐board sensors within mobile phones offer the ability to capture data remotely, without the need for specialized equipment. The increasing availability of mobile phone use is already supplementing existing forms of patient data, particularly in high‐income settings. In surgery, this presents exciting avenues for diagnosis and routine follow‐up, particularly in settings where patients cannot easily attend hospitals.

There are important areas of study that are more specific to resource‐poor areas, such as access to surgical care and the cost of surgical care to patients. Only one study^72^ was identified that explored the economic consequences of surgery; this reflected previous findings highlighting large‐scale health economic studies in cancer being focused in high‐income countries or heavily modelled using data from high‐income countries^103^, ^104^. Evaluating patient cost following surgery is likely to require frequent and long‐term follow‐up, potentially explaining the difficulties in measuring this outcome. Use of mobile technology to circumvent current logistical issues and capture expenditure data following surgery is an exciting avenue.

The landscape of healthcare data is changing rapidly. Ensuring that LMICs have the resources to keep up to date with technological advances will ensure future global health equality^10^. New developments, such as artificial intelligence, virtual reality, mobile computing and new molecular techniques, present exciting opportunities for surgeons across the world. Embedding these technologies within ‘learning’ healthcare systems will ensure that data contribute to the incremental development of safe practice. Big data are capable of providing information on safety, complications and survival; however, with the increasing use of big data, care must be taken to account for unknown and unrecognized confounders in order to determine intervention effectiveness and provide strong observational conclusions^105^.

In parallel with future advances, ensuring that electronic data are kept secure is of utmost importance. Respecting an individual patient's rights to confidentiality, autonomy and privacy is fundamental to ensuring public trust in electronic data collection methods. Beyond good data governance practice, technologies such as blockchain may facilitate the safe and secure sharing of healthcare data within increasingly complex interconnected systems.

There are weaknesses to the approach taken in this review. Pragmatic limitations around the scope of the review search were required and important studies may have been omitted. The synthesis of such a heterogenous group of studies is difficult and conclusions must be made at a high level.

This review has demonstrated a significant growth in the use of large‐volume patient‐level data across many surgical specialties and LMICs. At least 71 LMICs currently involved in big data projects were identified, with evidence of an exponential growth in patient numbers totalling more than 700 000. However, to date, the majority of studies using big data have been limited to short‐term outcomes after surgery and few have addressed the needs that are particular to LMICs. Funders, policymakers and specialists in medical informatics urgently need to reorientate this focus if the potential of big data to improve surgical outcomes, particularly in LMICs, is to be realized fully.

## Disclosure

The authors declare no conflict of interest.

## Supporting information

Table S1 Studies included within the systematic review
Table S2 Aims of included studies grouped by primary outcome measure and surgical specialty
