# Supplementary material for: Systematic review of the use of big data to improve surgery in low‐ and middle‐income countries
Source: Br J Surg. 2019 Jan 8;106(2):e62–72. doi: 10.1002/bjs.11052 (PMC6590290; doi:10.1002/bjs.11052)
Supplement: Supplementary file 1 — Table S1 Studies included within the systematic review Table S2 Aims of included studies grouped by primary outcome measure and surgical specialty [file BJS-106-e62-s001.docx]

**BJS11052**

**Systematic review of the use of big data to improve surgery in low- and middle-income countries**

S. R. Knight, R. Ots, M. Maimbo, T. M. Drake, C. J. Fairfield and E. M. Harrison

**Appendix S1 Systematic review literature search terms**

**EMBASE/Medline**

**1.** Developing Countries**.sh,kf.**

**2.** (Africa or Asia or Caribbean or West Indies or South America or Latin America or Central America)**.hw,kf,ti,ab,cp.**

**3.** (Afghanistan or Albania or Algeria or Angola or Armenia or Armenian or Azerbaijan or Bangladesh or Benin or Byelarus or Byelorussian or Belarus or Belorussian or Belorussia or Belize or Bhutan or Bolivia or Bosnia or Herzegovina or Hercegovina or Botswana or Brasil or Brazil or Bulgaria or Burkina Faso or Burkina Fasso or Upper Volta or Burundi or Urundi or Cambodia or Khmer Republic or Kampuchea or Cameroon or Cameroons or Cameron or Camerons or Cape Verde or Central African Republic or Chad or China or Colombia or Comoros or Comoro Islands or Comores or Mayotte or Congo or Zaire or Costa Rica or Cote d'Ivoire or Ivory Coast or Croatia or Cuba or Cyprus or Djibouti or French Somaliland or Dominica or Dominican Republic or East Timor or East Timur or Timor Leste or Ecuador or Egypt or United Arab Republic or El Salvador or Eritrea or Ethiopia or Fiji or Gabon or Gabonese Republic or Gambia or Gaza or Georgia Republic or Georgian Republic or Ghana or Gold Coast or Greece or Grenada or Guatemala or Guinea or Guam or Guiana or Guyana or Haiti or Honduras or India or Maldives or Indonesia or Iran or Iraq or Jamaica or Jordan or Kazakhstan or Kazakh or Kenya or Kiribati or Kosovo or Kyrgyzstan or Kirghizia or Kyrgyz Republic or Kirghiz or Kirgizstan or Lao PDR or Laos or Lebanon or Lesotho or Basutoland or Liberia or Libya or Lithuania or Macedonia or Madagascar or Malagasy Republic or Sabah or Sarawak or Malawi or Nyasaland or Mali or Marshall Islands or Mauritania or Mauritius or Agalega Islands or Mexico or Micronesia or Middle East or Moldova or Moldovia or Moldovian or Mongolia or Montenegro or Morocco or Ifni or Mozambique or Myanmar or Myanma or Burma or Namibia or Nepal or Netherlands Antilles or New Caledonia or Nicaragua or Niger or Nigeria or Northern Mariana Islands or Oman or Muscat or Pakistan or Palau or Palestine or Paraguay or Peru or Philippines or Philipines or Phillipines or Phillippines or Romania or Rumania or Roumania or Russia or Russian or Rwanda or Ruanda or Saint Kitts or St Kitts or Nevis or Saint Lucia or St Lucia or Saint Vincent or St Vincent or Grenadines or Samoa or Samoan Islands or Navigator Island or Navigator Islands or Sao Tome or Senegal or Serbia or Montenegro or Seychelles or Sierra Leone or Slovenia or Sri Lanka or Ceylon or Solomon Islands or Somalia or South Africa or Sudan or Suriname or Surinam or Swaziland or Syria or Tajikistan or Tadzhikistan or Tadjikistan or Tadzhik or Tanzania or Thailand or Togo or Togolese Republic or Tonga or Trinidad or Tobago or Tunisia or Turkey or Turkmenistan or Turkmen or Uganda or Ukraine or USSR or Soviet Union or Union of Soviet Socialist Republics or Uzbekistan or Uzbek or Vanuatu or New Hebrides or Venezuela or Vietnam or Viet Nam or West Bank or Yemen or Yugoslavia or Zambia or Zimbabwe or Rhodesia)**.hw,ti,ab,cp.**

**4.** ((developing or less* developed or under developed or underdeveloped or middle income or low* income or underserved or under served or deprived or poor*) adj (countr* or nation? or population? or world))**.ti,ab.**

**5.** ((developing or less* developed or under developed or underdeveloped or middle income or low* income) adj (economy or economies))**.ti,ab.**

**6.** (low* adj (gdp or gnp or gross domestic or gross national))**.ti,ab.**

**7.** (low adj3 middle adj3 countr*)**.ti,ab.**

**8.** (lmic or lmics or third world or lami countr*)**.ti,ab.**

**9.** transitional countr***.ti,ab.**

**10.** or/1-9

**11.** surgery or surg* not precision medicine

**12.** big data or large data or informatics or database or cohort or registry

**13.** Limits: Full text, English language, Humans, 2008 – present

**Table S1 Studies included within the systematic review**

| Author | Year of publication | Patient number | Included countries | Study design | Name of database / registry | Surgical specialty | Primary outcomes measured |
| --- | --- | --- | --- | --- | --- | --- | --- |
| Moghimi-Dehkordi et al^16^ | 2008 | 746 | Iran | Database | Cancer Registry Center of Research Center of Gastroenterology and Liver Disease (RCGLD) | General surgery | Post-operative outcomes |
| Zhaohui et al^17^ | 2008 | 1959 | China | Registry | Chinese Maxillofacial Trauma Registry | Maxillofacial surgery | Post-operative outcomes |
| Mariano et al^18^ | 2009 | 780 | Brazil | Database |  | Urology | Post-operative outcomes |
| Rezaianzadeh et al^19^ | 2009 | 1148 | Iran | Registry | Fars province registry, Southern Iran | Breast | Post-operative outcomes |
| Campos et al^20^ | 2009 | 4744 | Brazil | Registry |  | General surgery | Patient demographics / Post-operative outcomes |
| Elbasmi et al^21^ | 2010 | 902 | Kuwait | Registry vs Database | Kuwait Cancer Registry | Breast | Patient demographics |
| Biglarian et al^22^ | 2010 | 436 | Iran | Database | Cancer Archive of Taleghani Hospital | General surgery | Post-operative outcomes |
| Moghimi-Dehkordi et al^23^ | 2010 | 742 | Iran | Database | Taleghani hospital registry | General surgery | Post-operative outcomes |
| Chen et al^24^ | 2010 | 945 | China | Cohort |  | General surgery | Post-operative outcomes |
| Gupta et al^25^ | 2010 | 811 | India | Database |  | General | Incidence |
| ALANEPE (Latin American Pediatric Nephrology Association^26^ | 2010 | 1254 | Brazil (667), Chile (163), Venezuela (113), Mexico (171), Cuba (36), Colombia (17), Costa Rica (13), Nicaragua (9), Guatemala (3), Ecuador (3), Honduras (18), Paraguay (4) and Peru (5) | Registry |  | Paediatrics / Transplantation | Patient demographics / Post-operative outcomes |
| Muleta et al^27^ | 2010 | 14928 | Ethiopia | Cohort |  | Gynaecology | Patient demographics |
| Ercole et al^28^ | 2011 | 3543 | Brazil | Database | HRI database | Orthopaedics | Post-operative outcomes |
| Brisebois et al^29^ | 2011 | 4434 | Afghanistan | Database | Role 3 Multina- tional Medical Unit operating room database | Multiple specialties | Incidence |
| Ranasinghe et al^30^ | 2011 | 1378 | Sri Lanka | Registry | Sri Lanka cancer registry | Urology | Incidence |
| Nechuta et al^31^ | 2011 | 4877 | China | Registry + prospective follow-up | Shanghai Cancer Registry | Breast | Post-operative outcomes |
| Elias et al^32^ | 2011 | 625 | Brazil | Cohort |  | Multiple specialties | Post-operative outcomes |
| Frajzyngier et al^33^ | 2012 | 1273** | Uganda, Guinea, Niger, Nigeria, Bangladesh | Cohort |  | Obstetrics and gynaecology | Post-operative outcomes |
| Piotto et al^34^ | 2012 | 3010 | Brazil | Database |  | Cardiothoracics | Post-operative outcomes |
| Löfgren et al^35^ | 2014 | 563 | Uganda | Database |  | General surgery | Incidence |
| Tollefson et al^36^ | 2015 | 604 | Zimbabwe | Database |  | ENT | Patient demographics |
| Mejia et al^37^ | 2015 | 2565 | Brazil | Database |  | Cardiothoracics | Post-operative outcomes |
| Sivasubramaniam et al^38^ | 2015 | 4211 | China | Database | CICAMS | Breast surgery | Patient demographics |
| Noppakun et al^39^ | 2015 | 5729 | Thailand | Registry | Thai Transplant Registry | Transplantation | Patient demographics / Post-operative outcomes |
| SASOS^40^ | 2015 | 3927 | South Africa | Cohort |  | Multiple specialties | Patient demographics / Post-operative outcomes |
| Moodley et al^41^ | 2015 | 3727 | South Africa | Database |  | Multiple | Post-operative outcomes |
| Zablotska et al^42^ | 2015 | 11664 | Belarus | Database | BelAm | ENT | Incidence |
| Saifuddin et al^43^ | 2015 | 2198 | Pakistan | Cohort |  | Cardiothoracics | Post-operative outcomes |
| Filippi et al^44^ | 2015 | 950 | Burkina Faso | Cohort |  | Obstetrics and gynaecology | Post-operative outcomes / Quality of life |
| Sangkittipaiboon et al^45^ | 2015 | 1439 | Thailand | Registry | Lopburi registry | Breast | Incidence |
| Lalitwongsa et al^46^ | 2015 | 2458 | Thailand | Registry | Lampang cancer registry | Breast | Incidence |
| Tassanasunthornwong et al^47^ | 2015 | 1118 | Thailand | Registry | Surat Thani cancer registry | Breast | Incidence |
| Paula et al^48^ | 2015 | 2612 | Brazil | Database | SIH-SUS | Orthopaedics | Post-operative outcomes |
| Islam et al^49^ | 2015 | 3329 | Bangladesh | Database | SMPP database | Obstetrics and gynaecology | Incidence |
| Moreno et al^50^ | 2015 | 46539* | Europe | Database | EuSOS | Multiple specialties | Post-operative outcomes |
| ISOS^51^ | 2016 | 15806* | Worldwide | Cohort | ISOS | Multiple specialties | Post-operative outcomes |
| Nagoshi et al^52^ | 2016 | 479* | India, Turkey, Brazil, China, HIC | Cohort | AOSpine International Study | Spinal surgery | Post-operative outcomes / Quality of life |
| Tostes et al^53^ | 2016 | 3773 | Brazil | Database | Brazilian Medical Association | Multiple specialties | Access |
| Wang et al^54^ | 2016 | 2733 | China | Registry | Chinese liver transplant registry | Transplantation | Survival |
| Garcia et al^55^ | 2016 | 2506** | Nepal, India, Zambia | Database | NGO ReSurge International | Plastics | Patient demographics / Post-operative outcomes |
| Xiang et al^56^ | 2016 | 335 | China | Cohort |  | General Surgery | Post-operative outcomes / Patient survival |
| GlobalSurg collaborative^57^ | 2016 | 10745* | Worldwide | Cohort |  | Multiple specialties | Post-operative outcomes |
| Nandakumar et al^58^ | 2016 | 14053 | India | Cohort | National Centre for Disease Informatics and Research | Head and neck | Incidence / Post-operative outcomes |
| Reyes et al^59^ | 2016 | 2621 | Philippines | Registry | Eduardo J. Aboitiz Cancer Center Registry | General Surgery | Patient demographics |
| Lei et al^60^ | 2016 | 1004 | China | Cohort |  | General surgery | Patient demographics / Post-operative outcomes |
| Bhandare et al^61^ | 2017 | 580 | India | Cohort |  | General surgery | Survival |
| Acaroglu et al^62^ | 2017 | 535* | Turkey | Database |  | Spinal surgery | Post-operative outcomes |
| Fang et al^63^ | 2017 | 1183 | China | Database |  | General surgery | Survival |
| Figueiredo et al^64^ | 2017 | 2460 | Brazil | Cohort | BRAZPD II cohort | General surgery | Post-operative outcomes |
| Zheng et al^65^ | 2017 | 32040 | China | Registry | Chinese Cardiac Surgery Registry | Cardiothoracics | Post-operative outcomes |
| Yousefzadeh et al^66^ | 2017 | 588 | Iran | Registry |  | Paediatrics | Post-operative outcomes |
| Gajewski et al^67^ | 2017 | 27743 | Zambia (3549), Malawi (24194) | Cohort |  | Multiple specialties | Patient demographics |
| Shah et al^68^ | 2017 | 4143 | Nepal | Cohort |  | Multiple specialties | Patient demographics |
| Hu et al^69^ | 2017 | 3474 | China | Cohort | Ma’anshan Birth Cohort Study (MBCS) | Obstetrics and gynaecology | Patient demographics |
| Arthur et al^70^ | 2017 | 3285 | Brazil | Registry | Sao Paulo Registry of Cardiovascular Surgery (REPLICCAR) | Cardiothoracics | Post-operative outcomes |
| Hernandez et al^71^ | 2017 | 1415 | South Africa | Database | Pietermaritzburg Metropolitan Surgical Service | General Surgery | Patient demographics |
| The ACTION study group^72^ | 2017 | 9513 | Cambodia (206), Indonesia (2335), Lao PDR (101), Malaysia (1662), Myanmar (1178), Philippines (909), Thailand (1206), Vietnam (1916) | Cohort |  | Multiple specialties | Patient cost / Post-operative outcomes |
| Doshi et al^73^ | 2017 | 787 | India | Cohort |  | Orthopaedics | Post-operative outcomes |
| Kopp et al^74^ | 2017 | 346 | Malawi | Cohort |  | Gynaecology | Post-operative outcomes |
| Treeprasertsuk et al^75^ | 2017 | 34325 | Thailand | Database | Nationwide Hospital Admission Database | General Surgery | Incidence / Post-operative outcomes |
| Carvalho et al^76^ | 2017 | 16882 | Brazil | Cohort |  | General surgery | Post-operative outcomes |
| Wang et al^77^ | 2018 | 1977 | China | Database | Western China Clinical Cooperation Group | Breast surgery | Survival |
| van der Spuy et al^78^ | 2018 | 382 | South Africa | Cohort |  | Multiple specialties | Patient demographics |
| George et al^79^ | 2018 | 1075 | India | Cohort |  | Multiple specialties | Patient demographics |
| Augusto et al^80^ | 2018 | 428346 | Brazil | Registry | DATASUS | Gynaecology | Post-operative outcomes / Patient cost |
| Brand et al^81^ | 2018 | 3412 | South Africa | Database | Private healthcare funder's database | General surgery | Survival |
| ASOS (African Surgical Outcomes Study)^82^ | 2018 | 11422 | Congo, Dem. Rep. (315), Gambia, The (82), Madagascar (192), Mali (329), Mauritius (418), Namibia (325), Niger (186), Nigeria (395), South Africa (5522), Uganda (620), Zimbabwe (640), Algeria (184), Benin (220), Burundi (127), Cameroon (223), Congo (3), Egypt (10), Ethiopia (252), Ghana (225), Kenya (324), Libya (667), Senegal (7), Tanzania (97), Togo (19), Zambia (40) | Cohort |  | Multiple specialties | Post-operative outcomes |
| GlobalSurg collaborative^83^ | 2018 | 12539 | Worldwide | Cohort |  | Multiple specialties | Post-operative outcomes |

*Studies where country-specific numbers were not available

**Studies where total LMIC patient numbers were discernable

**Table S2 Aims of included studies grouped by primary outcome measure and surgical specialty**

| Primary outcome measure | Author | Year of publication | Patient number | Included countries | Surgical specialty | Article aim |
| --- | --- | --- | --- | --- | --- | --- |
| Patient demographics | Elbasmi et al^21^ | 2010 | 902 | Kuwait | Breast surgery | To determine reliability of Kuwait cancer registry |
|  | Sivasubramaniam et al^38^ | 2015 | 4211 | China | Breast surgery | Identify breast cancer disparities compared to American SEER database |
|  | Tollefson et al^36^ | 2015 | 604 | Zimbabwe | ENT | Estimate the burden of cleft lip-palate in Zimbabwe |
|  | *Campos et al^20^ | 2009 | 4744 | Brazil | General surgery | Evolution of laparoscopic colorectal surgery in Brazil |
|  | Reyes et al^59^ | 2016 | 2621 | Philippines | General Surgery | Colorectal cancer characteristics across metro Cebu district over 10 year period |
|  | *Lei et al^60^ | 2016 | 1004 | China | General surgery | Pathological data and prediction of microvascular invasion pre-operatively in patients with HCC |
|  | Hernandez et al^71^ | 2017 | 1415 | South Africa | General Surgery | Severity of appendicitis at presentation in South African cohort |
|  | Muleta et al^27^ | 2010 | 14928 | Ethiopia | Gynaecology | Presence of obstetric fistula across Ethiopia |
|  | Hu et al^69^ | 2017 | 3474 | China | Obstetrics and gynaecology | Measurement of placental response in low-risk childbirth |
|  | *Garcia et al^55^ | 2016 | 2506** | Nepal, India, Zambia | Plastics | Factors Affecting Burn Contracture Outcome in Developing Countries |
|  | *ALANEPE (Latin American Pediatric Nephrology Association^26^ | 2010 | 1254 | Multiple countries | Paediatrics / Transplantation | Latin American registry of paediatric renal transplantation |
|  | *Noppakun et al^39^ | 2015 | 5729 | Thailand | Transplantation | 25-year experience of kidney transplantation in Thailand |
|  | Gajewski et al^67^ | 2017 | 27743 | Zambia (3549), Malawi (24194) | Multiple specialties | Access to surgery in Sub-Saharan Africa |
|  | *SASOS^40^ | 2015 | 3927 | South Africa | Multiple specialties | 7-day surgical outcomes in South Africa |
|  | Shah et al^68^ | 2017 | 4143 | Nepal | Multiple specialties | Use of tablet elogbooks by 14 non-medical anaesthetic providers to measure complications |
|  | van der Spuy et al^78^ | 2018 | 382 | South Africa | Multiple specialties | Presence of hypertensive disease in elective surgery cohort |
|  | George et al^79^ | 2018 | 1075 | India | Multiple specialties | Myocardial injury after non-cardiac surgery |
| Incidence | Sangkittipaiboon et al^45^ | 2015 | 1439 | Thailand | Breast | Measurement of breast cancer incidence in Thailand district |
|  | Lalitwongsa et al^46^ | 2015 | 2458 | Thailand | Breast | Measurement of breast cancer incidence in Thailand district |
|  | Tassanasunthornwong et al^47^ | 2015 | 1118 | Thailand | Breast | Measurement of breast cancer incidence in Thailand district |
|  | Zablotska et al^42^ | 2015 | 11664 | Belarus | ENT | Malignant thyroid pathological findings following radiation exposure |
|  | Gupta et al^25^ | 2010 | 811 | India | General surgery | Presentation of renal tumours over 20 year period |
|  | Löfgren et al^35^ | 2014 | 563 | Uganda | General surgery | Prevalence of groin herniae in eastern Uganda |
|  | *Treeprasertsuk et al^75^ | 2017 | 34325 | Thailand | General Surgery | Burden and mortality of intrahepatic cholangiocarcinoma in Thailand |
|  | Islam et al^49^ | 2015 | 3329 | Bangladesh | Obstetrics and gynaecology | Rate of caesarean section in hospitals providing emergency obstetric care |
|  | Ranasinghe et al^30^ | 2011 | 1378 | Sri Lanka | Urology | Incidence of prostate cancer |
|  | Brisebois et al^29^ | 2011 | 4434 | Afghanistan | Multiple specialties | Multi-national medical unit experience in Afghanistan |
| Access | Tostes et al^53^ | 2016 | 3773 | Brazil | Multiple specialties | Access to surgical care in Brazil |
| Post-operative outcomes | Rezaianzadeh et al^19^ | 2009 | 1148 | Iran | Breast | Survival analysis of patients with breast cancer in Iran |
|  | Nechuta et al^31^ | 2011 | 4877 | China | Breast | Vitamin supplement use during breast cancer treatment |
|  | Piotto et al^34^ | 2012 | 3010 | Brazil | Cardiothoracics | Independent predictors of prolonged mechanical ventilation after coronary artery bypass surgery |
|  | Mejia et al^37^ | 2015 | 2565 | Brazil | Cardiothoracics | Age, creatinine and ejection fraction score in Brazil: Comparison with InsCor and the EuroSCORE |
|  | Saifuddin et al^43^ | 2015 | 2198 | Pakistan | Cardiothoracics | Developing cardiac surgery service in Pakistan |
|  | Zheng et al^65^ | 2017 | 32040 | China | Cardiothoracics | Comparing Outcomes of Coronary Artery Bypass Grafting among Large Teaching and Urban Hospitals |
|  | Arthur et al^70^ | 2017 | 3285 | Brazil | Cardiothoracics | Renal dysfunction following cardiac surgery |
|  | Nandakumar et al^58^ | 2016 | 14053 | India | Head and neck | Incidence / Post-operative outcomes |
|  | Moghimi-Dehkordi et al^16^ | 2008 | 746 | Iran | General surgery | Survival following gastric cancer resection |
|  | *Campos et al^20^ | 2009 | 4744 | Brazil | General surgery | Evolution of laparoscopic colorectal surgery in Brazil |
|  | Biglarian et al^22^ | 2010 | 436 | Iran | General surgery | Determining of prognostic factors in gastric cancer patients |
|  | Moghimi-Dehkordi et al^23^ | 2010 | 742 | Iran | General surgery | Impact of age on prognosis in Iranian patients with gastric carcinoma |
|  | Chen et al^24^ | 2010 | 945 | China | General surgery | Effect of diabetes mellitus on prognosis for patients undergoing resection for colorectal cancer |
|  | *Xiang et al^56^ | 2016 | 335 | China | General Surgery | Prospective cohort study of laparoscopic and open hepatectomy for hepatocellular carcinoma |
|  | *Lei et al^60^ | 2016 | 1004 | China | General surgery | Pathological data and prediction of microvascular invasion pre-operatively in patients with HCC |
|  | Figueiredo et al^64^ | 2017 | 2460 | Brazil | General surgery | Early exit site infection following peritoneal dialysis catheter insertion |
|  | *Treeprasertsuk et al^75^ | 2017 | 34325 | Thailand | General Surgery | Burden and mortality of intrahepatic cholangiocarcinoma in Thailand |
|  | Carvalho et al^76^ | 2017 | 16882 | Brazil | General surgery | Incidence and risk factors for surgical site infection in general surgery |
|  | Frajzyngier et al^33^ | 2012 | 1273** | Uganda, Guinea, Niger, Nigeria, Bangladesh | Obstetrics and gynaecology | Surgical methods for repair of genitourinary fistula |
|  | Filippi et al^44^ | 2015 | 950 | Burkina Faso | Obstetrics and gynaecology | The effects of life-saving caesarean sections in Burkina Faso |
|  | Kopp et al^74^ | 2017 | 346 | Malawi | Gynaecology | Continence status in women after obstetric vesicovaginal fistula repair |
|  | Augusto et al^80^ | 2018 | 428346 | Brazil | Gynaecology | Costs and mortality rates of surgical approaches to hysterectomy in Brazil |
|  | Ercole et al^28^ | 2011 | 3543 | Brazil | Orthopaedics | Risk of surgical site infection following orthopaedic surgery |
|  | Paula et al^48^ | 2015 | 2612 | Brazil | Orthopaedics | Readmission and death following hip fracture in elderly population |
|  | Doshi et al^73^ | 2017 | 787 | India | Orthopaedics | Infection following internal fixation of tibial fractures |
|  | Zhaohui et al^17^ | 2008 | 1959 | China | Maxillofacial surgery | Design and implementation of a maxillofacial trauma registry |
|  | *Garcia et al^55^ | 2016 | 2506** | Nepal, India, Zambia | Plastics | Factors Affecting Burn Contracture Outcome in Developing Countries |
|  | Nagoshi et al^52^ | 2016 | 479* | India, Turkey, Brazil, China, HIC | Spinal surgery | Outcomes following surgery for degenerative cervical myelopathy |
|  | Acaroglu et al^62^ | 2017 | 535* | Turkey | Spinal surgery | Surgery for adult spinal deformity |
|  | Yousefzadeh et al^66^ | 2017 | 588 | Iran | Paediatrics | Mortality, length of stay and surgery associated with paediatric trauma |
|  | *ALANEPE (Latin American Pediatric Nephrology Association^26^ | 2010 | 1254 | Multiple countries | Paediatrics / Transplantation | Latin American registry of paediatric renal transplantation |
|  | *Noppakun et al^39^ | 2015 | 5729 | Thailand | Transplantation | 25-year experience of kidney transplantation in Thailand |
|  | Mariano et al^18^ | 2009 | 780 | Brazil | Urology | Laparoscopic radical prostatectomy |
|  | Elias et al^32^ | 2011 | 625 | Brazil | Multiple specialties | Incidence and risk factors for sepsis in surgical patients |
|  | *SASOS^40^ | 2015 | 3927 | South Africa | Multiple specialties | 7-day surgical outcomes in South Africa |
|  | Moodley et al^41^ | 2015 | 3727 | South Africa | Multiple | Predictors of in-hospital mortality following non-cardiac surgery |
|  | Moreno et al^50^ | 2015 | 46539* | Europe | Multiple specialties | Utility of American Society of Anaesthesiologist score in predicting in-hospital mortality |
|  | ISOS^51^ | 2016 | 15806* | Worldwide | Multiple specialties | Global patient outcomes after elective surgery |
|  | GlobalSurg collaborative^57^ | 2016 | 10745* | Worldwide | Multiple specialties | Mortality of emergency abdominal surgery in high-, middle- and low-income countries |
|  | *The ACTION study group^72^ | 2017 | 9513 | Multiple countries | Multiple specialties | Policy and priorities for national cancer control planning in low- and middle-income countries |
|  | ASOS (African Surgical Outcomes Study)^82^ | 2018 | 11422 | Multiple countries | Multiple specialties | 7-day peri-operative patient outcomes in Africa |
|  | GlobalSurg collaborative^83^ | 2018 | 12539 | Worldwide | Multiple specialties | Surgical site infection following gastrointestinal surgery |
| Patient cost | *The ACTION study group^72^ | 2017 | 9513 | Multiple countries | Multiple specialties | Policy and priorities for national cancer control planning in low- and middle-income countries |
| Survival | Wang et al^77^ | 2018 | 1977 | China | Breast surgery | Predictors and survival in presence of internal mammary lymph nodes metastasis |
|  | *Xiang et al^56^ | 2016 | 335 | China | General Surgery | Prospective cohort study of laparoscopic and open hepatectomy for hepatocellular carcinoma |
|  | Bhandare et al^61^ | 2017 | 580 | India | General surgery | Radical gastrectomy in Indian tertiary centre |
|  | Fang et al^63^ | 2017 | 1183 | China | General surgery | Clinicopathologic characteristics and prognosis of gastroenteropancreatic neuroendocrine neoplasms |
|  | Brand et al^81^ | 2018 | 3412 | South Africa | General surgery | Long-term outcomes for colorectal cancer in South Africa |
|  | Wang et al^54^ | 2016 | 2733 | China | Transplantation | Preoperative sodium concentration, hepatitis B virus cirrhosis and liver transplantation |

*Studies where more than outcome was measured
